# Hydration- and Spacing-Governed Filtration Behavior of Cation-Exchanged Bentonites in Ca^2+^-Rich Brines

**DOI:** 10.3390/ma19081565

**Published:** 2026-04-14

**Authors:** Tian Xie, Mingliang Tang, Hai Zheng, Xiangwen Jiang, Chuanjiang Yang

**Affiliations:** 1 College of Materials Science and Engineering, Nanjing Tech University, Nanjing 211816, China; 15195832080@163.com (T.X.); 18862721868@163.com (X.J.); 18552438936@163.com (C.Y.); 2 Donghai Institute of Advanced Silicon-Based Materials, Nanjing Tech University, Lianyungang 222300, China; 15996130575@163.com

**Keywords:** cation-exchanged bentonite, CaCl_2_-rich brine, hydrated ionic radius, basal spacing, filtration behavior

## Abstract

Ca^2+^-rich brines strongly destabilize bentonite-based drilling fluids by weakening hydration and increasing filter-cake permeability. In this work, raw sodium bentonite (Na-Bt) and a series of cation-exchanged bentonites (Li-, Mg-, Ca-, and K-Bt) were comparatively investigated to clarify how cation-dependent hydration characteristics and interlayer structure govern filtration behavior under saline conditions. XRD, zeta potential, TG–DTG, BET, and SEM were employed to correlate basal spacing, surface electrostatic properties, thermal/water-loss behavior, surface area and pore-structure characteristics, and filter-cake microstructure with API fluid loss. Among the examined 2 wt% brines, CaCl_2_ produced the most severe deterioration and was therefore selected as the representative screening condition. Under 2 wt% CaCl_2_, Li-Bt exhibited the lowest *FL_API_* (141 mL), which was substantially lower than that of Na-Bt (265 mL), indicating the most favorable intrinsic resistance to Ca^2+^-dominated salinity. The cation-exchange analysis further showed that Li-Bt and Mg-Bt had relatively higher calculated exchange degrees than Ca-Bt and K-Bt under the present preparation conditions. Based on the 2 wt% CaCl_2_ dataset, a descriptor-based relation between *FL_API_*, hydrated ionic radius (r_h_), and basal spacing (d_001_) was established, and an Al-modified bentonite provided an out-of-sample verification with close agreement between predicted and measured filtration loss. Additional tests in 1–3 wt% CaCl_2_ showed that although absolute fluid loss increased with brine severity, the relative ranking of the cation-exchanged bentonites remained broadly unchanged. TG–DTG, BET, and SEM results further provided complementary evidence for the structural and microstructural differences among the samples. Overall, the results demonstrate that hydration-related response, interlayer structure, and surface/pore characteristics jointly govern the filtration behavior of cation-exchanged bentonites, providing a useful basis for screening salt-tolerant clay materials for Ca^2+^-rich brines.

## 1. Introduction

Bentonite is a typical layered clay material widely used in drilling fluids, barrier systems, and grouting suspensions because of its hydration, swelling, colloidal stability, and filtration-control properties [1,2]. Its macroscopic performance is mainly governed by the hydration state of montmorillonite layers, the nature of exchangeable interlayer cations, and the resulting particle interactions in aqueous environments [3]. However, these characteristics are highly sensitive to temperature and salinity. Under deep and ultra-deep drilling conditions, conventional sodium bentonite (Na-Bt) may suffer from weakened hydration, reduced dispersion stability, and the formation of more permeable filter cakes, resulting in deteriorated rheological performance and increased filtrate invasion [4,5].

Among saline contaminants, divalent-ion-rich brines are generally more destructive than monovalent salt systems because they more effectively compress the electrical double layer, weaken hydration repulsion, and promote particle association [6,7]. In particular, Ca^2+^-containing brines can severely destabilize bentonite suspensions by altering interlayer hydration, reducing swelling ability, and accelerating structural deterioration under thermal aging [8,9]. Although the harmful effects of salinity on bentonite-based drilling fluids are well known, the selection of salt-tolerant bentonites remains largely empirical [10]. Therefore, establishing a rational screening framework for bentonite materials in Ca^2+^-dominated saline environments is important.

Various strategies have been proposed to improve the salt tolerance of bentonite-based fluids, including polymer treatment, nanoparticle incorporation, organic modification, and multi-component additives [11,12,13]. However, these approaches often increase compositional complexity and may obscure the intrinsic response of the clay itself. In contrast, cation exchange directly modifies the interlayer environment of montmorillonite and thus affects hydration strength, swelling behavior, surface electrostatic characteristics, and particle packing [14,15]. Nevertheless, fewer studies have established a physically interpretable relation between cation-dependent structural characteristics and macroscopic filtration behavior under a defined Ca^2+^-rich brine condition [16].

Another important issue is that many screening concepts are established at a single salinity level, whereas their applicability within a practical salinity window remains unclear [17]. Although a relation derived at one CaCl_2_ concentration may be useful for ranking materials under that condition, its applicability under weaker or more severe CaCl_2_ contamination still needs to be evaluated. Therefore, extending the assessment from a fixed 2 wt% CaCl_2_ condition to a 1–3 wt% CaCl_2_ concentration window is meaningful for testing the robustness and applicability boundary of the screening relation.

Therefore, in this work, Na-Bt and a series of cation-exchanged bentonites (Li-, Mg-, Ca-, and K-Bt) were comparatively investigated to clarify how cation-dependent hydration characteristics and interlayer structure govern filtration behavior in Ca^2+^-rich brines. X-ray diffraction (XRD) and zeta potential measurements were employed to characterize the structural and surface changes induced by cation exchange, while API filtration tests, concentration-window evaluation in 1–3 wt% CaCl_2_, and scanning electron microscopy (SEM) observations of representative filter cakes were combined to establish the relation between microscopic descriptors and macroscopic filtration response. The objectives of this study are threefold: (i) to identify the most stringent brine condition among several common salt systems; (ii) to establish a CaCl_2_-specific structure–property screening relation linking hydrated ionic radius and basal spacing to filtration behavior; and (iii) to assess the robustness and applicability boundary of this screening concept across a practical CaCl_2_ concentration window. The originality of this work lies in linking the filtration behavior of cation-exchanged bentonites under a defined Ca^2+^-rich brine condition to experimentally accessible structural descriptors, rather than evaluating salt resistance only in an empirical manner. In addition, by extending the analysis from a fixed 2 wt% CaCl_2_ condition to a broader 1–3 wt% CaCl_2_ concentration window, this study provides a more practically relevant assessment of the robustness and applicability boundary of the proposed screening framework. Therefore, this work not only advances the mechanistic understanding of how hydration characteristics and interlayer structural response jointly govern bentonite filtration behavior but also offers a useful basis for the rational screening of salt-tolerant bentonite materials for Ca^2+^-dominated saline environments.

## 2. Materials and Methods

### 2.1. Raw Materials and Reagents

Sodium bentonite (Na-Bt), obtained from Guangxi, China, was used as the starting clay material for all modification and performance tests. Magnesium chloride (analytical grade, 99.0%, Shanghai Macklin Biochemical Co., Ltd., Shanghai, China), calcium chloride (analytical grade, 99.9%, Shanghai Macklin Biochemical Co., Ltd., Shanghai, China), potassium chloride (analytical grade, 99.5%, Shanghai Macklin Biochemical Co., Ltd., Shanghai, China), and lithium chloride (analytical grade, 99.0%, Shanghai Macklin Biochemical Co., Ltd., Shanghai, China) were used as ion-exchange reagents for the preparation of cation-exchanged bentonites. Hydrochloric acid (ACS specification, 37%, Merck KGaA, Darmstadt, Germany) was used in the preparation of Li-exchanged bentonite. Ultrapure water was used throughout the ion-exchange and sample preparation procedures. The hydrated ionic radii of the exchange cations were taken from the literature [18] and used as descriptor variables in the subsequent structure–property analysis.

### 2.2. Preparation of Cation-Exchanged Bentonites

Na-Bt was converted into Mg-, Ca-, and K-exchanged bentonites by aqueous ion exchange using the corresponding chloride salts. Briefly, the chloride salt was dissolved in ultrapure water, followed by the addition of Na-Bt. The resulting slurry was stirred at 60 °C and 1000 rpm for 6 h to allow ion exchange. After reaction, the solid phase was separated by filtration, dried at 105 °C, ground, and sieved to obtain the corresponding cation-exchanged bentonite powders, denoted as Mg-Bt, Ca-Bt, and K-Bt, respectively.

**Figure 1:**
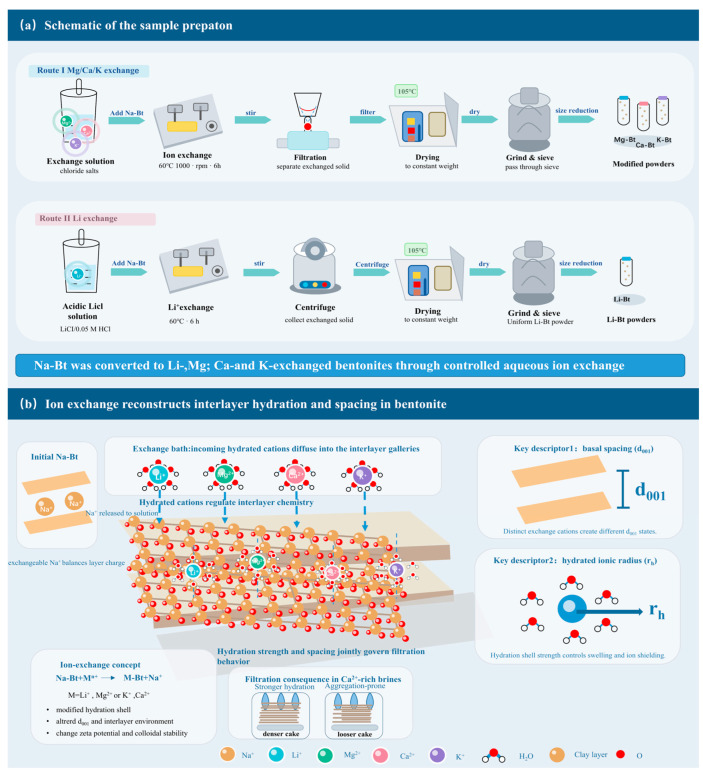
(**a**) Schematic of the sample preparation (**b**) Schematic of ion exchange.

Li-exchanged bentonite (Li-Bt) was prepared using a modified aqueous exchange route. LiCl was first dissolved in 0.05 M HCl, and Na-Bt was then added at a solid-to-liquid ratio of 1.5–2.5:1. The suspension was stirred at 60 °C for 6 h. After reaction, the solids were collected by centrifugation, followed by drying at 105 °C, grinding, and sieving to obtain Li-Bt. The overall preparation route and the cation-exchange concept are illustrated schematically in Figure 1a,b.

### 2.3. Characterization of Cation-Exchanged Bentonites

The structural and surface-property changes induced by cation exchange were characterized by X-ray diffraction (XRD) and zeta potential measurements. XRD was used to analyze the basal spacing (d_001_) of the raw bentonite and the cation-exchanged bentonites (Li-Bt, Mg-Bt, Ca-Bt, and K-Bt), in order to evaluate the effect of different exchange cations on the interlayer structure of montmorillonite. Zeta potential measurements were performed to assess the changes in surface electrostatic properties after cation exchange. All samples were tested under identical conditions to ensure the comparability of the results. The hydrated ionic radii of the exchange cations, together with the measured basal spacing values, were further used as key descriptors in the subsequent structure–property correlation analysis. In addition to the structural and surface characterization described above, the cation-exchange characteristics summarized in Table 1 were also evaluated using the parent sodium bentonite (Na-Bt) as the reference. The cation exchange capacity (CEC) of the parent Na-Bt was determined from the amount of exchangeable interlayer Na^+^ in the sodium-form bentonite and expressed as cmol(+)/kg. For the cation-exchanged bentonites, the residual Na content was determined from the Na content remaining after ion-exchange treatment, while the introduced cation content was obtained from the content of the corresponding exchange cation in the exchanged samples. Both residual Na and introduced cation contents were expressed as mmol/100 g. To compare the exchange extent among different cation-exchanged bentonites, the introduced cation contents were converted to a charge-equivalent basis and compared with the CEC of the parent Na-Bt. The estimated *exchange degree* was calculated as follows:

(1) $$Exchange\;degree\;\left(\%\right)=\frac{C_{introduced}\times Z}{{CEC}_{parent}}\times 100$$

where *C_introduced_* is the introduced cation content (mmol/100 g), *Z* is the cation valence, and *CEC_parent_* is the CEC of the parent Na-Bt expressed in the same equivalent-charge unit. For monovalent cations, *Z* = 1, and for divalent cations, *Z* = 2. Because 1 cmol(+)/kg is numerically equivalent to 1 mmol(+)/100 g, the converted introduced-cation contents could be directly compared with the parent CEC. The residual Na content was used as an auxiliary indicator of incomplete replacement.

XRD measurements were carried out using a D2 PHASER X-ray diffractometer (Bruker, Karlsruhe, Germany) with Cu Kα radiation (λ = 1.5406 Å). Prior to testing, the powder samples were dried at 105 °C to constant weight, ground, passed through a 200-mesh sieve, and mounted by the tablet-pressing method to minimize the influence of preferred orientation on peak shape. The diffraction patterns were collected over a 2θ range of 5–90° at a scanning rate of 10°/min. Phase identification was carried out using Jade 6 software with the PDF 2006 database. The main phases identified in the bentonite samples included montmorillonite, quartz, and calcite. The phase assignment was based on matching the experimental diffraction patterns with the corresponding entries in the PDF 2006 database and was further checked against the relevant literature. In the present study, particular attention was paid to the (001) reflection of montmorillonite for determining the basal spacing (d_001_) variation after cation exchange. The basal spacing (d_001_) values of the bentonite samples were determined from the corresponding (001) diffraction peak positions using Bragg’s law.

Thermogravimetric analysis (TGA) was further conducted on the representative Li-Bt sample to evaluate its thermal behavior and water-loss characteristics. The measurement was carried out using a TG 209 thermogravimetric analyzer (NETZSCH, Selb, Germany) under a nitrogen atmosphere. Before heating, nitrogen was introduced for at least 5 min to ensure an inert environment in the furnace. The sample was then heated at a rate of 10 °C/min, and the corresponding TG–DTG curves were recorded. The obtained results were used to provide supplementary information on the hydration-related water loss and thermal behavior of the modified bentonite.

Brunauer–Emmett–Teller (BET) surface area analysis was further conducted on Na-Bt and the cation-exchanged bentonites (Li-Bt, Mg-Bt, Ca-Bt, and K-Bt) to characterize their surface area and pore-structure-related features. Nitrogen adsorption–desorption measurements were carried out on a BSD-660M surface area and porosity analyzer (Beishide Instrument Technology (Beijing) Co., Ltd., Beijing, China) using the static volumetric method at 77.3 K. Prior to analysis, the samples were degassed under vacuum at 110 °C for 90 min. The specific surface area was calculated from the adsorption isotherms using the BET method. These results were used as supplementary structural evidence for discussing the differences in surface accessibility, pore characteristics, and filtration behavior among the bentonite samples.

Zeta potential measurements were performed using a Zetasizer Nano Z analyzer (Malvern Panalytical, Malvern, UK). The bentonite samples were dispersed in deionized water, and the pH of the suspensions was adjusted to 7 prior to testing. This pH was selected as a unified comparison condition for all samples, rather than to directly simulate the alkaline chemistry of practical drilling fluids. All measurements were conducted at 25 °C. Each sample was measured in triplicate, and the average value was reported. Raw bentonite and the modified samples were comparatively analyzed under the same dispersion conditions to evaluate the effect of cation exchange on the surface electrostatic characteristics.

Scanning electron microscopy (SEM) was further employed to examine the microstructure of representative filter cakes, so as to clarify the microstructural origin of the differences in filtration behavior. According to the filtration ranking under CaCl_2_-rich conditions, representative filter cakes of Na-Bt and Li-Bt were selected for observation. SEM analysis was conducted using a JSM-7800F field-emission scanning electron microscope (JEOL, Tokyo, Japan). After the API filtration tests, the filter cakes were carefully peeled off, dried at 105 °C for 16 h, and the central region of each cake was selected to minimize edge effects. The dried samples were mounted on conductive adhesive and sputter-coated with gold prior to imaging. SEM images were acquired at an accelerating voltage of 15 kV, and a magnification of 50,000× was used as the principal comparison condition. Particular attention was paid to the stacking mode of clay platelets, pore size and connectivity, flocculation/aggregation features, and crack development, in order to correlate the filter-cake microstructure with the observed filtration behavior.

### 2.4. Preparation and Aging of Bentonite-Based Drilling Fluids

Bentonite-based drilling fluids were prepared using the raw bentonite and the cation-exchanged bentonites as the solid-phase materials. For all formulations, the clay concentration was fixed at 6 wt% to ensure comparability among samples. To evaluate salt tolerance under saline conditions, the bentonite slurries were prepared directly in brine rather than by adding salt after hydration in fresh water. Specifically, the required amount of bentonite powder was dispersed directly into the corresponding salt solution and mixed according to the same procedure for all samples, so that the effect of cation exchange on the intrinsic response to salinity could be compared under identical preparation conditions.

To identify the most stringent brine condition, a series of bentonite-based drilling fluids were prepared in different 2 wt% brines, including NaCl, KCl, MgCl_2_, and CaCl_2_. Based on the comparative rheological and filtration results, CaCl_2_ was identified as the representative worst-case brine condition for the subsequent screening and descriptor-based analysis. Additional concentration-window tests were further carried out in 1–3 wt% CaCl_2_ to evaluate the filtration response of the cation-exchanged bentonites under progressively worsening Ca^2+^-rich conditions and to assess the applicability boundary of the fixed 2 wt% CaCl_2_ screening framework.

For thermal-aging evaluation, the prepared drilling fluids were aged using an XGRL-4A high-temperature roller heating furnace (Qingdao Haitongda Special Instrument Co., Ltd., Qingdao, China). The samples were sealed in aging cells and hot-rolled at 180 °C for 16 h to simulate the salt–thermal coupled downhole environment. After aging, the fluid samples were cooled to room temperature prior to subsequent rheological and filtration measurements. The same preparation and aging procedure was applied to all bentonite systems to ensure the consistency of the comparative analysis.

### 2.5. Rheological and Filtration Measurements Under Saline Conditions

The rheological behavior and filtration performance of the bentonite-based drilling fluids were evaluated before and after thermal aging under saline conditions. Apparent viscosity (*AV*), plastic viscosity (*PV*), yield point (*YP*), the *YP*/*PV* ratio, and API fluid loss (*FL_API_*) were used as the principal evaluation parameters to assess the structural stability and filtration-control ability of the raw and cation-exchanged bentonite systems. Rheological measurements were conducted using a six-speed rotational viscometer, and the dial readings at 600 and 300 rpm were denoted as *θ*_600_ and *θ*_300_, respectively. Based on the Bingham plastic model, the rheological parameters were calculated as follows:

(2) $$AV=\frac{\theta_{600}}{2}$$

(3) $$PV=\theta_{600}-\theta_{300}$$

(4) $$YP=\theta_{300}-PV$$

where *AV* reflects the overall flow resistance and structural development of the bentonite suspension, *PV* represents the viscous contribution arising from interparticle and particle–liquid friction under flow, and *YP* characterizes the structural strength associated with particle interactions and network formation. To further evaluate the balance between structural carrying capacity and viscous resistance, the *YP*/*PV* ratio was also used:

(5) $$b=\frac{YP}{PV}$$

A higher *YP/PV* ratio generally indicates a stronger structural contribution relative to viscous resistance; however, under saline conditions, an increased ratio may also arise from salt-induced flocculation and should therefore be interpreted together with *AV*, *PV*, and the filtration results.

To identify the most severe saline environment, comparative rheological and filtration tests were first performed in several 2 wt% brines, including NaCl, KCl, MgCl_2_, and CaCl_2_. Based on the deterioration of both rheological stability and filtration control, CaCl_2_ was identified as the most stringent brine condition and was therefore selected as the representative screening environment for the subsequent analysis. Detailed rheological and filtration evaluations were then carried out under 2 wt% CaCl_2_. In addition, concentration-window tests in 1–3 wt% CaCl_2_ were further performed to assess the response regularity of the cation-exchanged bentonites under progressively worsening Ca^2+^-rich conditions and to evaluate the practical robustness of the fixed 2 wt% CaCl_2_ screening framework.

Filtration performance under ambient conditions was evaluated using the API fluid-loss test, and the filtrate volume collected within 30 min was recorded as the API fluid loss, *FL_API_* (mL). A lower *FL_API_* generally indicates the formation of a denser and less permeable filter cake, corresponding to better filtration-control performance. The measured *FL_API_* values were used as the principal macroscopic response for ranking the salt resistance of Na-Bt, Li-Bt, Mg-Bt, Ca-Bt, and K-Bt under different saline conditions. After filtration, representative filter cakes were collected for subsequent SEM observation in order to correlate cake compactness and microstructural features with the measured filtration performance. In particular, Na-Bt and Li-Bt were selected as representative samples for filter-cake comparison under the base-fluid condition, 2 wt% CaCl_2_ contamination, and the salt–thermal coupled condition of 180 °C + 2 wt% CaCl_2_.

To ensure comparability, all rheological and filtration measurements were conducted using the same preparation, aging, and testing procedure for the raw and cation-exchanged bentonite systems. The *FL_API_* values obtained under 2 wt% CaCl_2_ were subsequently used in the descriptor-based analysis linking hydrated ionic radius and basal spacing to filtration behavior, while the results obtained in the 1–3 wt% CaCl_2_ concentration window were further used to evaluate the applicability boundary of the fixed 2 wt% CaCl_2_ screening relation.

## 3. Results

### 3.1. Structural and Surface Characteristics of Cation-Exchanged Bentonites

To clarify how cation exchange modified the intrinsic properties of bentonite, the structural and surface characteristics of Na-Bt and the cation-exchanged bentonites were first analyzed by XRD and zeta potential measurements [19,20]. Particular attention was paid to the basal spacing (d_001_) and the surface electrostatic properties, because these parameters are closely related to interlayer hydration, colloidal stability, and the subsequent filtration behavior under saline conditions [21,22].

Table 1 summarizes the cation-exchange characteristics of the parent Na-Bt and the corresponding modified bentonites.

Based on the introduced-cation content and the CEC of the parent Na-Bt, Li-Bt and Mg-Bt exhibited relatively higher calculated exchange degrees than Ca-Bt and K-Bt, suggesting more effective replacement of interlayer Na^+^ under the present preparation conditions.

**Figure 2:**
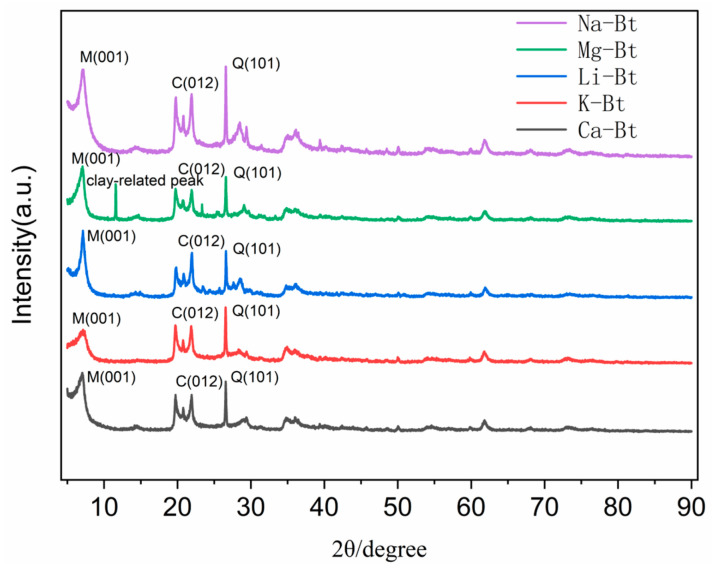
XRD patterns of raw bentonite (Na-Bt) and cation-exchanged bentonites (Mg-Bt, Li-Bt, K-Bt, and Ca-Bt). The main reflections were labeled according to the identified phases, where *M* denotes montmorillonite, *Q* denotes quartz, and *C* denotes calcite. The weak feature at approximately 12° (2θ) in the Mg-Bt diffractogram was marked as a clay-related peak for clarity.

As shown in Figure 2, the XRD patterns of Na-Bt and the cation-exchanged bentonites exhibited noticeable differences in the position of the (001) reflection, indicating that the interlayer structure of montmorillonite was altered after cation exchange [23,24]. The main reflections were labeled according to the identified phases, and the weak peak at approximately 12° (2*θ*) in the Mg-Bt diffractogram was marked as a clay-related reflection for clarity.

In addition to the montmorillonite reflections, the XRD pattern of the raw Na-Bt showed the presence of accessory quartz and calcite, indicating that the starting bentonite was a natural multi-mineral material. Nevertheless, montmorillonite was identified as the dominant clay phase and was therefore taken as the principal structural phase for evaluating the effect of cation exchange. Since the purpose of the present XRD analysis was to track the cation-dependent variation of the montmorillonite (001) reflection, the following discussion focuses mainly on the change in basal spacing. The shift of the montmorillonite (001) reflection in Figure 2 indicates that the basal spacing (d_001_) changed after cation exchange. The d_001_ value of the raw bentonite was 1.2415 nm, whereas those of Mg-Bt, Li-Bt, Ca-Bt, and K-Bt were 1.2500, 1.2550, 1.2672, and 1.2445 nm, respectively. Compared with Na-Bt, the cation-exchanged bentonites generally showed enlarged basal spacing, although the magnitude of the change depended on the type of exchange cation. The largest d_001_ was observed for Ca-Bt, while K-Bt showed only a slight increase. These results indicate that different exchangeable cations produced distinct interlayer structural states. According to previous XRD, experimental, and simulation studies, exchangeable cations strongly control the organization of interlayer water and ions in smectites, thereby affecting their hydration/swelling and colloidal dispersion behavior under saline conditions [25,26]. Therefore, the different interlayer states identified here likely play a role in the subsequent filtration response of the bentonite systems.

**Figure 3:**
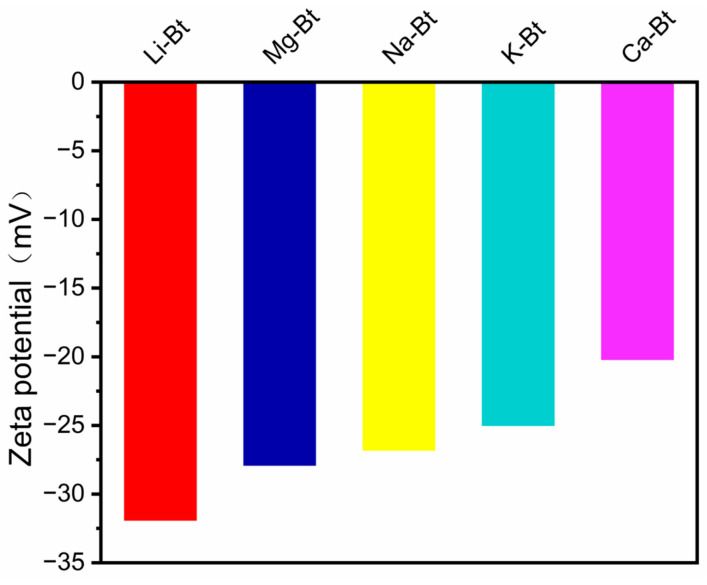
Zeta potentials of Na-Bt and the cation-exchanged bentonites under identical dispersion conditions.

As shown in Figure 3, the zeta potentials of Li-Bt, Mg-Bt, Na-Bt, K-Bt, and Ca-Bt were different after cation exchange, indicating that different exchange cations imposed distinct surface electrostatic characteristics on the bentonite particles.

The zeta potential results further confirmed that cation exchange altered the surface electrostatic properties of the bentonite particles [27,28]. As shown in Figure 3, the absolute values of zeta potential followed the order of Li-Bt (−31.9 mV) > Mg-Bt (−27.9 mV) > Na-Bt (−26.8 mV) > K-Bt (−25.0 mV) > Ca-Bt (−20.2 mV). Compared with Na-Bt, Li-Bt and Mg-Bt exhibited more negative zeta potentials, whereas K-Bt and especially Ca-Bt showed less negative values. These differences indicate that different exchange cations generated distinct surface electrostatic characteristics and therefore different levels of colloidal stability in aqueous media. In general, a larger absolute value of zeta potential corresponds to stronger electrostatic repulsion between particles, which is favorable for maintaining dispersion stability and resisting salt-induced aggregation [29,30]. Together with the XRD results, these findings demonstrate that cation exchange modified both the interlayer structural state and the surface electrostatic behavior of bentonite, thereby providing the material basis for the distinct rheological stability and filtration behavior observed under subsequent saline conditions.

Overall, the XRD and zeta potential results consistently indicate that cation exchange modified both the interlayer structural state and the surface electrostatic characteristics of bentonite. These changes are expected to influence the hydration, dispersion stability, and salt-resistance of the bentonite systems, thereby providing the material basis for the distinct rheological and filtration behaviors discussed in the following sections.

### 3.2. Thermal Behavior of Li-Bt

**Figure 4:**
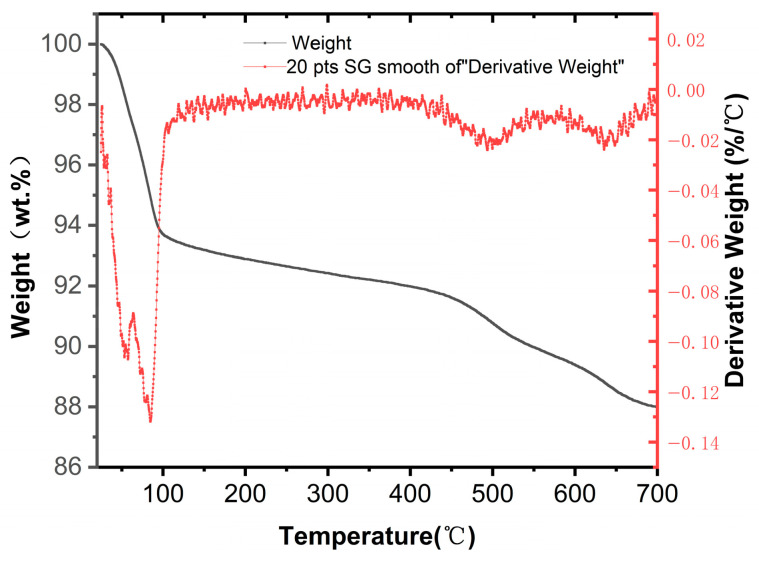
TG–DTG curves of the representative Li-Bt sample.

TG–DTG analysis was performed on the representative Li-Bt sample to examine its thermal behavior and hydration-related water-loss characteristics. The corresponding TG–DTG curves are shown in Figure 4.

As shown in Figure 4, the Li-Bt sample exhibited a characteristic two-stage weight-loss behavior during heating. The first major mass-loss region occurred below approximately 100 °C, where the sample weight decreased rapidly from about 100 wt.% to approximately 93.5 wt.%, accompanied by a pronounced DTG peak. This process is mainly attributed to the removal of physically adsorbed water and interlayer water, indicating the presence of hydration-related water in the Li-Bt system. Therefore, the TG–DTG result provides supplementary evidence for the existence of interlayer water in the modified bentonite. Above 100 °C, the mass decreased more gradually, suggesting that no further substantial release of weakly bound water occurred in this temperature range. A second, relatively mild mass-loss process appeared in the range of about 450–700 °C, which is attributed to the dehydroxylation of structural hydroxyl groups and thermally induced structural changes in the clay mineral. This high-temperature mass-loss behavior provides additional information on the thermal response and structural stability of Li-Bt. Overall, the TG–DTG results provide supplementary information on the hydration-related water loss and thermal behavior of Li-Bt, thereby supporting the interpretation of its structural and filtration behavior in combination with the other characterization results.

### 3.3. BET Surface Area and Pore-Structure Characteristics of Cation-Exchanged Bentonites

**Figure 5:**
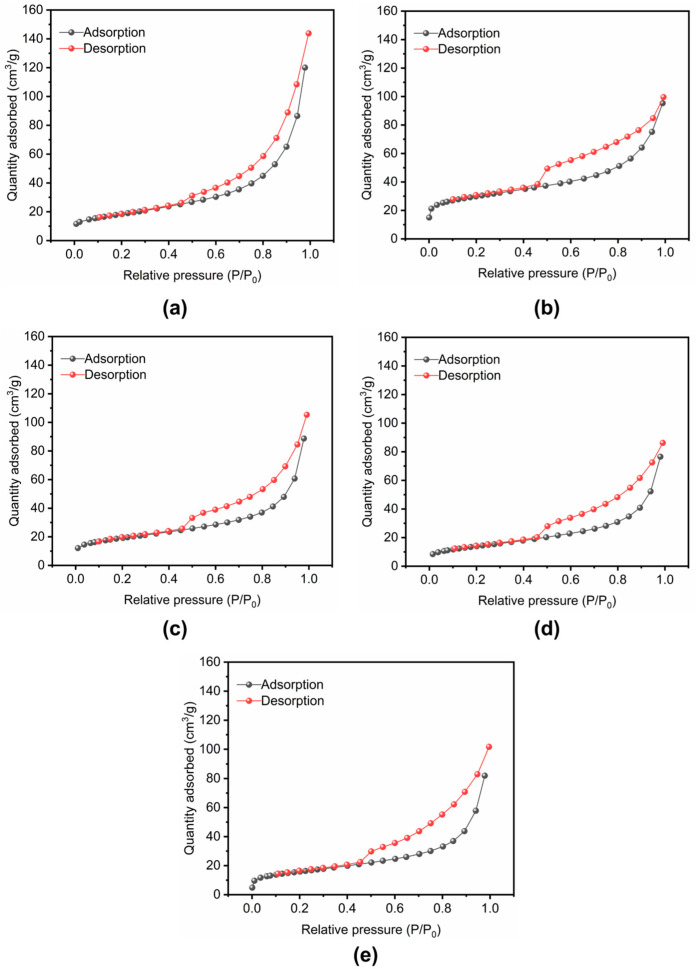
N_2_ adsorption–desorption isotherms used for BET surface area analysis of raw bentonite (Na-Bt) and cation-exchanged bentonites: (**a**) Na-Bt, (**b**) Mg-Bt, (**c**) Li-Bt, (**d**) Ca-Bt, and (**e**) K-Bt.

N_2_ adsorption–desorption (BET) analysis was conducted on Na-Bt and the cation-exchanged bentonites to characterize their surface area and pore-structure-related features, and the corresponding isotherms are shown in Figure 5.

As shown in Figure 5, all samples exhibited evident N_2_ adsorption–desorption hysteresis behavior, indicating the presence of mesopore-related structural features in the bentonite systems. The calculated specific surface areas were 65.3082 m^2^/g for Na-Bt, 67.6756 m^2^/g for Mg-Bt, 66.9441 m^2^/g for Li-Bt, 48.6303 m^2^/g for Ca-Bt, and 55.1810 m^2^/g for K-Bt. These results indicate that cation exchange affected the accessible surface area and pore-structure characteristics of bentonite to different extents. In particular, Li-Bt and Mg-Bt exhibited relatively higher specific surface areas than Ca-Bt and K-Bt, whereas Ca-Bt showed the lowest value. Such differences are consistent with cation-dependent variations in aggregation state and filter-cake packing behavior. Nevertheless, BET surface area alone cannot fully explain the filtration ranking, and the observed fluid-loss behavior should be understood as the combined result of interlayer structure, hydration-related behavior, surface electrostatic properties, and microstructural packing characteristics. Therefore, the BET analysis serves as complementary structural evidence supporting the interpretation of the filtration behavior of the cation-exchanged bentonites.

### 3.4. Salt-Specific Deterioration and Identification of CaCl_2_ as the Stringent Brine Condition

**Figure 6:**
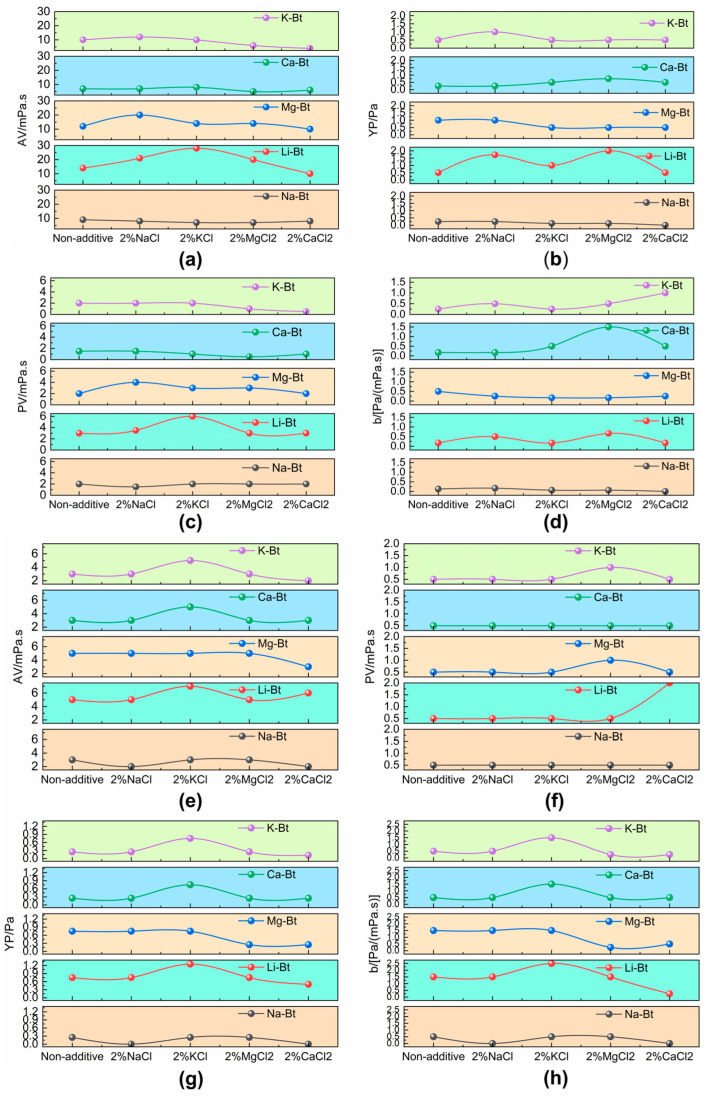
Rheological responses of raw bentonite (Na-Bt) and cation-exchanged bentonites (Li-Bt, Mg-Bt, Ca-Bt, and K-Bt) in different 2 wt% brines (NaCl, KCl, MgCl_2_, and CaCl_2_) before and after thermal aging. Panels (**a**–**d**) show the rheological parameters of the freshly prepared drilling fluids before aging: (**a**) apparent viscosity (*AV*), (**b**) yield point (*YP*), (**c**) plastic viscosity (*PV*), and (**d**) *YP*/*PV* ratio. Panels (**e**–**h**) show the corresponding rheological parameters after thermal aging at 180 °C for 16 h: (**e**) *AV*, (**f**) *PV*, (**g**) *YP*, and (**h**) *YP*/*PV* ratio. All samples were prepared under identical formulation conditions, and the comparison was used to evaluate the relative severity of different brine environments.

To identify the most severe saline environment for bentonite-based drilling fluids, comparative rheological tests were first carried out in several 2 wt% brines, including NaCl, KCl, MgCl_2_, and CaCl_2_. The raw bentonite and the cation-exchanged bentonites were evaluated under identical preparation, aging, and testing conditions, and the changes in apparent viscosity (*AV*), plastic viscosity *(PV*), yield point (*YP*), and *YP/PV* ratio were compared to determine the relative severity of different salt systems. For clarity, Figure 6a–d show the rheological parameters of the freshly prepared drilling fluids before thermal aging, whereas Figure 6e–h show the corresponding rheological parameters after aging at 180 °C for 16 h.

As shown in Figure 6, the bentonite-based drilling fluids exhibited different rheological response patterns in the presence of different brines, both before and after thermal aging. Compared with the monovalent salt systems, the divalent-ion-containing brines caused more pronounced deterioration in rheological stability. Among the tested brines, CaCl_2_ produced the most severe adverse effect, as evidenced by the larger changes in *AV*, *PV*, *YP*, and *YP*/*PV*. This result indicates that Ca^2+^-rich brine imposed the strongest destabilizing effect on the bentonite systems, which can be attributed to its stronger compression of the electrical double layer and its greater tendency to weaken interparticle hydration repulsion and promote aggregation. Therefore, CaCl_2_ was identified as the representative stringent brine condition for the subsequent screening and descriptor-based analysis of the cation-exchanged bentonites.

More specifically, Figure 6a and Figure 6e show the variations in *AV* before and after thermal aging, respectively, indicating that the overall flow resistance and structural development of the bentonite suspensions were strongly affected by brine type. Figure 6b,g show the corresponding *YP* values before and after aging, reflecting the salt-dependent change in particle-interaction strength and network structure. Figure 6c,f present the *PV* variations before and after aging, showing the effect of different brines on viscous resistance arising from interparticle and particle–liquid friction under flow. Figure 6d,h show the *YP*/*PV* ratio before and after aging, further illustrating the salt-dependent balance between structural contribution and viscous resistance. In all cases, the NaCl and KCl systems were relatively less destructive, MgCl_2_ showed an intermediate effect, and CaCl_2_ caused the most pronounced rheological deterioration.

Overall, Figure 6 indicates that the rheological response of bentonite was highly dependent on brine type, and this trend is consistent with the stronger destabilizing effect of divalent cations, especially Ca^2+^, on the hydration and dispersion stability of bentonite suspensions.

### 3.5. Filtration Ranking of Cation-Exchanged Bentonites Under 2 wt% CaCl_2_

**Figure 7:**
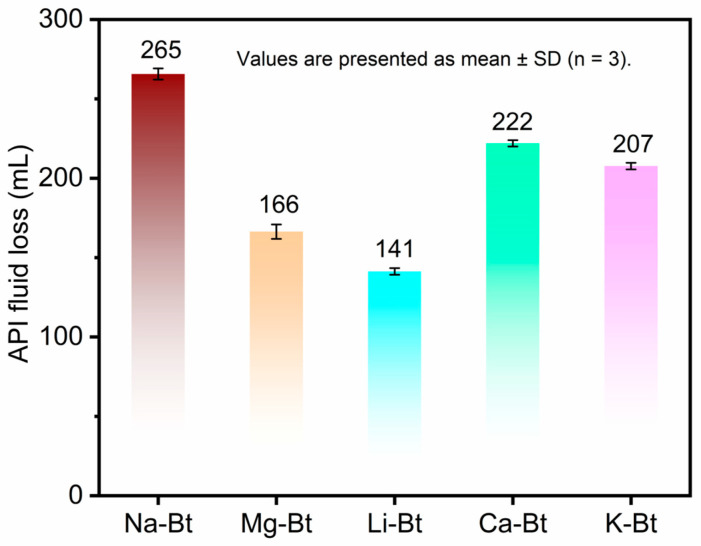
API fluid loss (*FL_API_*) of raw bentonite (Na-Bt) and cation-exchanged bentonites (Li-Bt, Mg-Bt, Ca-Bt, and K-Bt) under the representative stringent saline condition of 2 wt% CaCl_2_**.** This figure specifically presents the filtration-performance ranking of the bentonite samples under the selected CaCl_2_ condition.

To further clarify the filtration performance of different cation-exchanged bentonites under the representative stringent saline condition, the API fluid loss of Na-Bt, Li-Bt, Mg-Bt, Ca-Bt, and K-Bt was comparatively evaluated in 2 wt% CaCl_2_. The corresponding results are shown in Figure 7.

As shown in Figure 7, the cation-exchanged bentonites exhibited distinct filtration performances under the representative stringent condition of 2 wt% CaCl_2_. The values are presented as mean ± standard deviation (*n* = 3), and the error bars indicate good reproducibility of the measurements. Li-Bt showed the lowest *FL_API_* value (141 mL), indicating the best filtration-control performance among the tested samples. In contrast, Na-Bt exhibited the highest fluid loss (265 mL), while Mg-Bt, Ca-Bt, and K-Bt showed intermediate values of 166, 222, and 207 mL, respectively.

These results demonstrate that cation exchange significantly affected the resistance of bentonite to Ca^2+^-rich salinity, and that Li^+^ exchange was the most favorable for reducing filtration loss under the selected condition. The relatively small standard deviations compared with the differences among the samples further support the reliability of the observed filtration ranking.

### 3.6. Descriptor-Based Relation Under 2 wt% CaCl_2_ and Its Practical Verification

**Figure 8:**
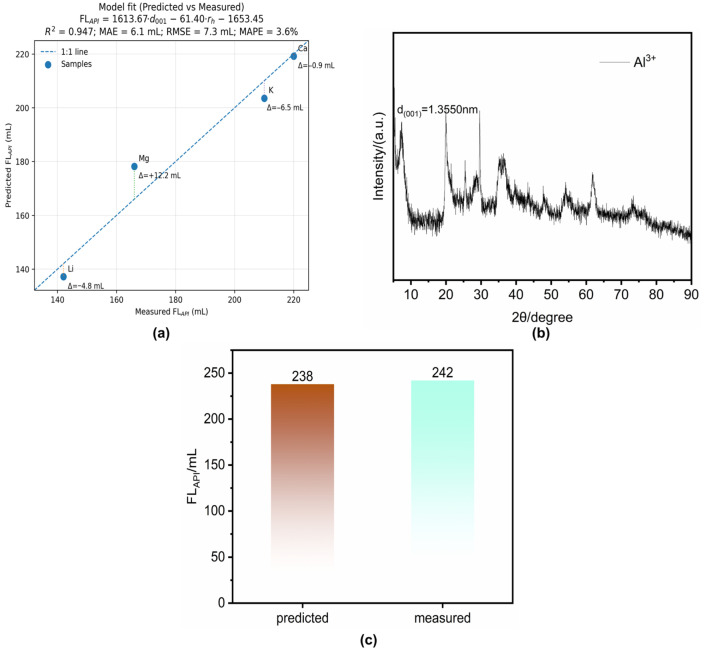
(**a**) Predicted versus measured FL_API_ values for the CaCl_2_-specific descriptor-based screening relation. (**b**) XRD pattern of Al-modified bentonite used for out-of-sample verification. (**c**) Comparison between the predicted and measured *FL_API_* values of the Al-modified bentonite under the 2 wt% CaCl_2_ condition.

To further interpret the cation-dependent filtration ranking under 2 wt% CaCl_2_, a descriptor-based analysis was subsequently carried out using hydrated ionic radius (r_h_) and basal spacing (d_001_). By restricting the analysis to the CaCl_2_ dataset only, the interference arising from cross-salt differences in ion speciation and governing mechanisms could be avoided. The corresponding results are shown in Figure 8.

As shown in Figure 8a, the predicted and measured *FL_API_* values exhibited close agreement for the cation-exchanged bentonites under the 2 wt% CaCl_2_ condition, indicating that the descriptor-based relation can reasonably reproduce the filtration ranking within the selected dataset. Based on the 2 wt% CaCl_2_ data only, an empirical screening relation was established as *FL_API_* = 1613.67·d_001_ − 61.40·r_h_ − 1653.45 (R^2^ = 0.947), linking the macroscopic fluid-loss response to two experimentally accessible descriptors, namely hydrated ionic radius and basal spacing. As an additional verification, Al-modified bentonite was introduced into the same framework. Its XRD pattern is shown in Figure 8b, and the predicted and measured *FL_API_* values were 238 and 242 mL, respectively (Figure 8c), showing close agreement. These results suggest that the CaCl_2_-specific relation can serve as a practical empirical screening framework for modified bentonites under the same brine condition. However, it should be regarded as a condition-specific empirical relation rather than a universal predictive model, and its applicability should be limited to the selected CaCl_2_ environment.

To further assess the robustness and applicability boundary of the fixed 2 wt% CaCl_2_ screening relation, the filtration responses of the cation-exchanged bentonites were further examined across a broader CaCl_2_ concentration window.

### 3.7. Salinity-Window Response and Applicability Boundary of the Fixed 2 wt% Screening Relation

**Figure 9:**
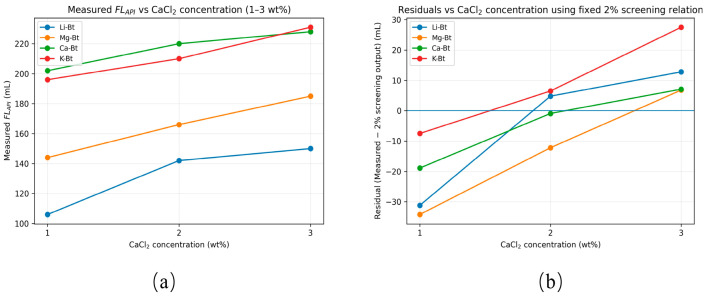
Salinity-window robustness test (1–3 wt% CaCl_2_) and residual analysis based on the fixed 2 wt% screening relation. (**a**) Measured *FL_API_* of Li-, Mg-, Ca- and K-exchanged bentonites as a function of CaCl_2_ concentration under identical formulation/aging/API filtration conditions. (**b**) Residuals relative to the fixed 2 wt% screening-relation output.

To evaluate the robustness of the fixed 2 wt% CaCl_2_ screening relation, the filtration responses of Li-Bt, Mg-Bt, Ca-Bt, and K-Bt were further examined in a 1–3 wt% CaCl_2_ concentration window under identical formulation, aging, and API filtration conditions. The corresponding results are shown in Figure 9.

As shown in Figure 9a, the measured *FL_API_* values of all cation-exchanged bentonites increased with increasing CaCl_2_ concentration, indicating that filtration control deteriorated progressively as the brine severity increased. Nevertheless, the relative ranking among the tested bentonites remained broadly consistent across the salinity window, with Li-Bt maintaining the lowest fluid-loss level throughout the 1–3 wt% CaCl_2_ range. This result suggests that the cation-dependent filtration ranking established at 2 wt% CaCl_2_ retained practical relevance within a bounded concentration window.

The residual analysis shown in Figure 9b further indicates that the fixed 2 wt% screening relation produced systematic deviations when applied to off-design salinity conditions. At 1 wt% CaCl_2_, the measured *FL_API_* values were generally lower than the values implied by the fixed 2 wt% screening output, whereas at 3 wt% CaCl_2_, the deviations shifted in the opposite direction. These results indicate that the descriptor-based relation should be regarded as a condition-specific empirical screening framework for the selected 2 wt% CaCl_2_ environment, rather than a universal predictive equation independent of salinity.

Overall, the salinity-window test confirms that the fixed 2 wt% CaCl_2_ relation is practically useful for ranking the relative salt resistance of cation-exchanged bentonites within Ca^2+^-dominated brines, while its quantitative applicability remains bounded by the selected concentration condition.

### 3.8. Microstructural Origin of the Filtration Behavior

**Figure 10:**
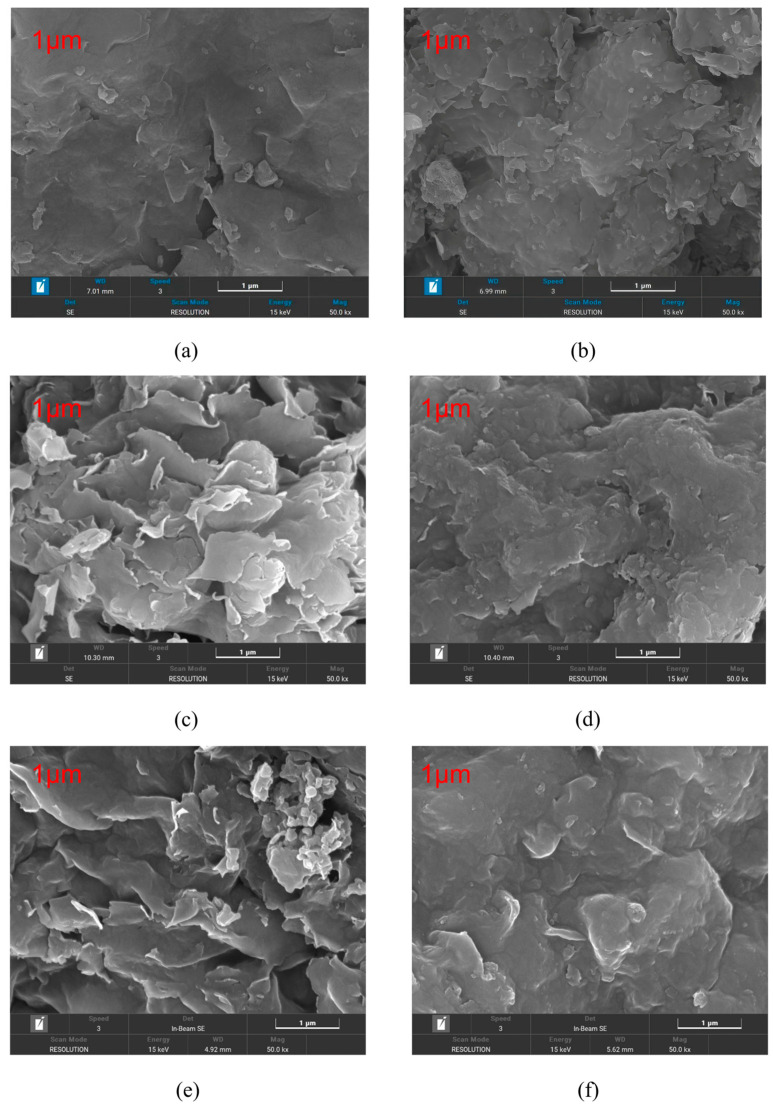
Original SEM micrographs of representative filter cakes formed by Na-Bt and Li-Bt under different conditions: (**a**) Na-Bt under the base-fluid condition; (**b**) Li-Bt under the base-fluid condition; (**c**) Na-Bt under 2 wt% CaCl_2_; (**d**) Li-Bt under 2 wt% CaCl_2_; (**e**) Na-Bt under 180 °C + 2 wt% CaCl_2_; and (**f**) Li-Bt under 180 °C + 2 wt% CaCl_2_. The magnification, scale bar, and imaging conditions are indicated in each panel.

To further elucidate the microstructural origin of the observed filtration ranking, representative filter cakes were examined by SEM under the base-fluid condition, the 2 wt% CaCl_2_ condition, and the salt–thermal coupled condition of 180 °C + 2 wt% CaCl_2_. According to the filtration results, Na-Bt and Li-Bt were selected as representative samples for comparison. The corresponding SEM images are shown in Figure 10a–f.

As shown in Figure 10a,b, both Na-Bt and Li-Bt formed relatively continuous filter cakes under the base-fluid condition, but Li-Bt exhibited a more compact platelet packing and a more homogeneous microstructure. After exposure to 2 wt% CaCl_2_ (Figure 10c,d), the filter cake of Na-Bt became looser and more porous, whereas Li-Bt still maintained a comparatively denser and less permeable structure. Under the salt–thermal coupled condition of 180 °C + 2 wt% CaCl_2_ (Figure 10e,f), the structural deterioration of Na-Bt became more severe, as indicated by larger pores, looser stacking, and more obvious discontinuities in the cake structure. In contrast, Li-Bt still retained a relatively compact and continuous microstructure, although some degree of structural degradation was also observed.

As shown by the SEM images, Li-Bt formed a denser and more homogeneous filter cake than Na-Bt under both saline and salt–thermal coupled conditions, whereas Na-Bt tended to form a looser and more permeable cake microstructure. These microstructural differences are consistent with the filtration ranking observed in the preceding sections. This interpretation is also in line with previous studies showing that exchangeable cations strongly affect the hydration behavior and interlayer structural response of bentonite/smectite, which in turn influence the compactness and permeability-related behavior of clay systems in saline environments [16].

### 3.9. Discussion

The results suggest that the filtration behavior of the cation-exchanged bentonites under CaCl_2_-rich conditions is controlled by the combined action of interlayer structure, hydration-related response, and surface electrostatic properties. XRD showed that different exchange cations produced distinct basal spacings, while the zeta potential results indicated clear differences in colloidal stability among the samples. In particular, Li-Bt and Mg-Bt exhibited more negative zeta potentials than Ca-Bt and K-Bt, implying stronger electrostatic repulsion and a lower tendency toward salt-induced aggregation. These differences are consistent with the view that cation exchange modifies both the interlayer structural state and the dispersion behavior of bentonite, which in turn affect the ability of the particles to form an effective sealing structure under saline conditions.

The TG–DTG, BET, and SEM results further support this interpretation from the perspectives of water state, accessible surface structure, and filter-cake morphology. TG–DTG confirmed the presence of hydration-related water in Li-Bt, while BET showed that cation exchange changed the accessible surface area and pore-structure-related characteristics of the samples. SEM observations finally revealed that Li-Bt formed a denser and more homogeneous filter cake than Na-Bt, whereas Na-Bt tended to form a looser and more porous structure under saline and salt–thermal coupled conditions. Therefore, the lower fluid loss of Li-Bt is more reasonably attributed to a more favorable combination of hydration-related stability, surface electrostatic behavior, and microstructural packing, rather than to any single structural parameter alone.

## 4. Conclusions

In this work, the filtration behavior of raw bentonite and cation-exchanged bentonites in saline environments was systematically investigated, with particular emphasis on CaCl_2_-rich brines. The results showed that cation exchange modified the interlayer structural state, surface electrostatic characteristics, thermal/water-loss behavior, and surface area/pore-structure-related features of bentonite, thereby influencing its hydration, dispersion stability, and filtration response. The calculated exchange-degree results further indicated that Li-Bt and Mg-Bt achieved relatively higher Na^+^ replacement levels than Ca-Bt and K-Bt under the present preparation conditions. Among the tested 2 wt% brines, CaCl_2_ caused the most severe deterioration in rheological stability and filtration control and was therefore identified as the representative stringent brine condition. Under 2 wt% CaCl_2_, Li-Bt exhibited the lowest API fluid loss (141 mL), indicating the best filtration-control performance among the tested samples. A CaCl_2_-specific empirical screening relation linking *FL_API_* with hydrated ionic radius and basal spacing was established and further supported by verification using Al-modified bentonite. The salinity-window test in 1–3 wt% CaCl_2_ confirmed that, although the absolute fluid loss increased with increasing CaCl_2_ concentration, the relative filtration ranking of the cation-exchanged bentonites remained broadly consistent. TG–DTG and BET analyses provided supplementary structural evidence for hydration-related water state, thermal behavior, accessible surface area, and pore-structure differences, while SEM observations showed that Li-Bt formed a denser and more homogeneous filter cake than Na-Bt under both saline and salt–thermal coupled conditions. Overall, the results demonstrate that cation-dependent hydration characteristics and interlayer structural response jointly govern the filtration behavior of bentonite in CaCl_2_-rich brines, providing a practical basis for screening salt-tolerant bentonite materials. These findings also provide useful guidance for the selection and design of bentonite-based materials for drilling-fluid and related applications in Ca^2+^-rich saline environments.

## Figures and Tables

**Table 1:** Cation-exchange characteristics of Na-Bt and cation-exchanged bentonites.

Sample	Dominant Interlayer Cation	CEC of Parent Na-Bt (cmol(+)/kg)	Residual Na (mmol/100 g)	Introduced Cation Content (mmol/100 g)	Estimated Exchange Degree (%)
Na-Bt	Na^+^	84.7 ± 1.3	84.7	0.0	0
Li-Bt	Li^+^	84.7 ± 1.3	10.7	74	87
Mg-Bt	Mg^2+^	84.7 ± 1.3	12.6	36.05	85
Ca-Bt	Ca^2+^	84.7 ± 1.3	16.1	34.3	80
K-Bt	K^+^	84.7 ± 1.3	15.3	69.4	81

## Data Availability

The original contributions presented in this study are included in the article. Further inquiries can be directed to the corresponding author.
